# Mild behavioral impairment-apathy and Alzheimer's disease plasma phosphorylated tau biomarker levels

**DOI:** 10.1177/13872877261470257

**Published:** 2026-07-29

**Authors:** Daniella Vellone, Rebeca Leon, Zahra Goodarzi, Nils D. Forkert, Eric E. Smith, Zahinoor Ismail

**Affiliations:** 1 Faculty of Graduate Studies, 2129 University of Calgary, Calgary, AB, Canada; 2 Hotchkiss Brain Institute, 70401 Cumming School of Medicine, University of Calgary, Calgary, AB, Canada; 3 Mathison Centre for Mental Health Research and Education, 70401 Cumming School of Medicine, University of Calgary, Calgary, AB, Canada; 4 Department of Community Health Sciences, 70401 Cumming School of Medicine, University of Calgary, Calgary, AB, Canada; 5 Department of Medicine, 70401 Cumming School of Medicine, University of Calgary, Calgary, AB, Canada; 6 O'Brien Institute for Public Health, 70401 Cumming School of Medicine, University of Calgary, Calgary, AB, Canada; 7 Department of Clinical Neurosciences, 70401 Cumming School of Medicine, University of Calgary, Calgary, AB, Canada; 8 Department of Radiology, 70401 Cumming School of Medicine, University of Calgary, Calgary, AB, Canada; 9 Alberta Children's Hospital Research Institute, 2129 University of Calgary, Calgary, AB, Canada; 10 Department of Psychiatry, 70401 Cumming School of Medicine, University of Calgary, Calgary, AB, Canada; 11 Department of Pathology and Laboratory Medicine, 2129 University of Calgary, Calgary, AB, Canada; 12 Clinical and Biomedical Sciences, Faculty of Health and Life Sciences, 3286 University of Exeter, Exeter, UK

**Keywords:** Alzheimer's disease, apathy, behavioral and psychological symptoms of dementia, biomarkers, cognitive dysfunction, dementia, mild behavioral impairment, neuropsychiatric symptoms, plasma

## Abstract

**Background:**

Mild behavioral impairment (MBI), characterized by later-life emergence of persistent neuropsychiatric symptoms (NPS), is an early clinical indicator of dementia risk. Global MBI has been associated with Alzheimer's disease (AD) pathology; studies have also explored MBI domains. Prior work has linked MBI-apathy to AD cerebrospinal fluid (CSF) biomarkers, but whether associations are detectable using plasma-based biomarkers such as phosphorylated tau (p-tau) is unknown. Establishing such relationships is critical, as plasma biomarkers are more accessible than CSF.

**Objective:**

To explore cross-sectional and longitudinal associations between MBI-apathy and plasma p-tau_181_ levels using Alzheimer's Disease Neuroimaging Initiative data.

**Methods:**

Older adults with normal cognition or mild cognitive impairment were categorized as MBI-apathy (n = 69), non-MBI NPS (n = 112), and no-NPS (n = 215) based on Neuropsychiatric Inventory scores and symptom persistence over one year. Linear regression modelled cross-sectional associations between NPS group and plasma p-tau_181_, adjusting for age, sex, education, apolipoprotein E4 status, and Mini-Mental State Examination score. Hierarchical linear mixed-effects modelling assessed associations over two and three years, including time-by-NPS group interactions.

**Results:**

MBI-apathy was associated with significantly higher plasma p-tau_181_ levels at baseline (24.05% [6.06–45.08%]; adjusted *p* = 0.014), and over two (26.46% [7.24–49.12%]; adjusted *p* = 0.012) and three years (29.28% [10.17–51.72%]; adjusted *p* = 0.004) compared to no-NPS. No significant associations were observed for non-MBI NPS. In sensitivity analyses, non-MBI apathy was not associated with plasma p-tau_181_ at baseline (−9.96% [−32.69–20.44%]; unadjusted *p* = 0.478).

**Conclusions:**

MBI-apathy is associated with elevated plasma p-tau_181_ cross-sectionally and longitudinally, supporting MBI-apathy as a potential proxy marker of tau pathology for early AD detection.

## Introduction

Alzheimer's disease (AD) is a progressive neurodegenerative condition characterized by the accumulation of pathological tau and amyloid-β proteins (Aβ).^1^ Biomarker-based criteria are now central to the diagnosis and staging of AD, shifting the field towards incorporating AD-specific biology into what was primarily a clinical definition of the disease.^1^ In the Alzheimer's Association 2024 revised AD criteria,^1^ the biological definition of AD is grounded in a two-tiered biomarker classification system for diagnosis and staging. Core 1 biomarkers are essential for diagnosis and include measures of amyloid and tau. Specifically, these markers include A for Aβ (i.e., cerebrospinal fluid [CSF] and blood plasma Aβ_42_, as well as amyloid positron emission tomography [PET]) and T1 for phosphorylated tau [p-tau], (i.e., CSF and blood plasma p-tau_181/217/231_). In contrast, Core 2 biomarkers are not required for diagnosis but can support staging and further biological characterization. Core 2 biomarkers reflect later-stage tau pathology (T2), including microtubule-binding region tau_243_, other phosphorylated tau forms (e.g., p-tau_205_), non-phosphorylated mid-region tau fragments, and tau PET imaging.

One of the earliest detectable stages of AD pathophysiology in biomarker-based models is marked by amyloid accumulation.^2^ As soluble Aβ is sequestered into plaques, CSF Aβ_42_ levels decline, and amyloid PET signal increases, often years before the onset of clinical symptoms.^3–8^ Following amyloid plaque deposition, tau becomes aberrantly phosphorylated at multiple mid-region residues, reflecting downstream effects of Aβ-driven kinase dysregulation and glial activation.^1,3–5,9^ Distinct phospho-epitopes, including threonine 181 (p-tau_181_) and threonine 217 (p-tau_217_), show overlapping but temporally distinct trajectories.^3–5^ P-tau_217_ rises earliest and more steeply with emerging Aβ pathology, whereas p-tau_181_ increases slightly later and plateaus more gradually during the prodromal phase.^10^ Phosphorylation at threonine 181 likely represents an early but downstream response to amyloid plaque formation, preceding the development of insoluble tau aggregates detectable by tau PET.^1,5,11^ Consequently, phosphorylated tau species function as intermediate markers linking amyloid deposition to later neurodegenerative changes. Although p-tau_217_ has demonstrated superior sensitivity and specificity for detecting early AD pathology,^3,11–17^ p-tau_181_ remains a valuable biomarker for longitudinal designs, as its trajectory levels are known to increase across preclinical and prodromal stages. The reliable detectability of p-tau_181_ in plasma enhances scalability for early identification of AD pathophysiology, especially in settings where access to neuroimaging or CSF collection is limited. The integration of fluid biomarkers into diagnostic workflows represents a pivotal shift toward earlier and more efficient identification of individuals at risk.

Although cognitive symptoms have traditionally provided the impetus for AD assessment and work up, neuropsychiatric symptoms (NPS) are recognized as early manifestations of the disease. In particular, mild behavioral impairment (MBI) is a validated framework for dementia detection and prognostication that leverages risk associated with later-life emergent and persistent NPS. Multiple studies have demonstrated that MBI is associated with incident cognitive decline and dementia.^18–22^ MBI comprises five behavioral domains, including apathy, affective dysregulation, impulse dyscontrol, social inappropriateness, and psychosis, some of which have also been studied longitudinally for risk of incident cognitive decline and dementia.^23–26^ Among these domains, apathy has consistently shown particularly strong associations with clinical progression and AD-related pathology.^24,27^ Apathy in neurocognitive disorders is defined as diminished interest, initiative, and/or emotional reactivity,^28–31^ and is particularly salient given its strong associations with tau pathology and progression to AD dementia.^24,27^ In earlier work, we demonstrated that MBI-apathy was associated with greater risk of incident dementia, even in the absence of cognitive impairment.^24^ More recently, we found that MBI-apathy is associated with greater CSF tau pathology.^27^ These findings support a domain-specific, hypothesis-driven focus on apathy as a behavioral phenotype closely linked to AD-related pathology. Few studies, however, have examined whether these associations extend to plasma-based biomarkers in non-dementia populations.^32,33^ Clarifying the relationship between MBI-apathy and plasma biomarkers is important, as these biomarkers are less invasive and more scalable than CSF or imaging, making them well suited for real-world clinical implementation. As MBI-apathy is a clinically observable and easily screenable behavioral syndrome, establishing alignment with plasma tau pathology could provide a pragmatic avenue for early risk stratification in pre-dementia populations.

Accordingly, the objective of this study was to investigate associations between MBI-apathy and plasma p-tau_181_ in individuals who are cognitively normal (CN) or have mild cognitive impairment (MCI). We hypothesized that MBI-apathy is associated with elevated plasma p-tau_181_ levels at baseline and that this association persists longitudinally over two- and three-year follow-up periods.

## Methods

### Study cohort and data source

This study utilized data from the Alzheimer's Disease Neuroimaging Initiative (ADNI; https://adni.loni.usc.edu/), a large, multicenter, longitudinal study launched in 2003 under the leadership of Principal Investigator Michael W. Weiner, MD. Supported by both public and private funding, ADNI was established to develop standardized clinical, imaging, genetic, and biomarker methodologies for assessing individuals across the cognitive spectrum, from normal cognition to mild AD. The dataset includes comprehensive demographic, diagnostic, neurological, genetic, and neuropathological assessments collected at six- to twelve-month intervals. ADNI adheres to Good Clinical Practice guidelines, the ethical principles of the Declaration of Helsinki, and U.S. regulatory requirements (21 CFR Part 50: Protection of Human Subjects and Part 56: Institutional Review Boards). The study protocol was approved by the Institutional Review Boards at all participating sites. All participants provided written informed consent for participation and publication prior to the initiation of any study procedures. Participant privacy was protected through the use of coded research identifiers; no personally identifying information was linked to the data used in this analysis. Data and samples were collected from September 2005 to September 2023. The available ADNI data used in this work were accessed and downloaded on March 11, 2024.

### Participant inclusion criteria and classification

**Figure 1.**
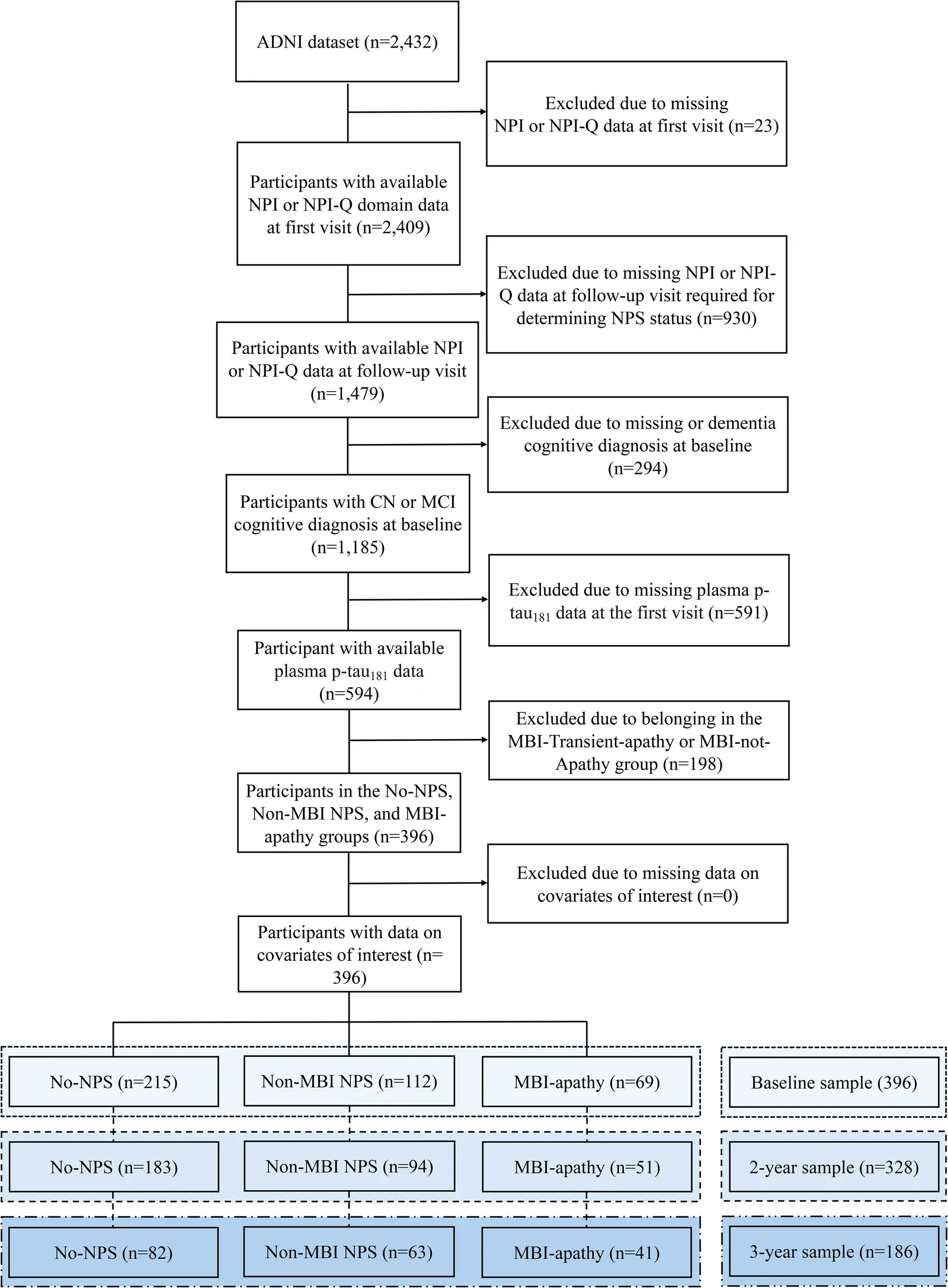
Flowchart of participants from ADNI included for analysis. ADNI: Alzheimer's Neuroimaging Initiative; CN: cognitively normal; MBI: mild behavioral impairment; MCI: mild cognitive impairment; NPI: neuropsychiatric inventory; NPI-Q: Neuropsychiatric Inventory Questionnaire; NPS: neuropsychiatric symptoms.

The study flow for primary data analysis is shown in Figure 1. Cognitive diagnoses (CN or MCI) were based on the Mini-Mental State Examination (MMSE) and Clinical Dementia Rating (CDR). CN participants had an MMSE score ≥24 and a CDR score of 0. Participants with MCI had an MMSE score ≥24, a CDR score of 0.5, memory complaints, and objective memory impairment based on education-adjusted scores from the Wechsler Memory Scale Logical Memory II, with preserved activities of daily living.

The classification of participant groups in this study follows a previously established methodology.^27^ Participants were included if they had complete Neuropsychiatric Inventory (NPI)^34^ (from ADNI-2, 3) or Neuropsychiatric Inventory Questionnaire (NPI-Q)^35^ (from ADNI-1, GO, 2, 3) domain scores necessary for determining NPS status and at least two study visits within their first year to assess symptom persistence.

As previously described,^27^ MBI status was determined by transforming NPI and NPI-Q scores into MBI domain scores using a published algorithm.^36^ Since the NPI and NPI-Q assess NPS over the previous four weeks, rather than over the ≥6-month period required for MBI, multiple study visits were used to approximate the MBI persistence criterion of ≥6 months. MBI-apathy was operationalized based on the NPI and NPI-Q apathy domain scores. The presence of apathy at a given visit was defined as an apathy domain score >0. To meet MBI-apathy criteria, apathy had to be present at two or more of the three visits within the first year (i.e., 0 and 6 months, 0 and 12 months, 6 and 12 months, or all three time points). Individuals were included in the MBI-apathy group irrespective of concurrent NPS in other MBI domains. Participants who met MBI criteria without apathy (Non-apathy MBI) or who exhibited apathy at only one visit but met MBI criteria in another domain (MBI-transient-apathy), were excluded from primary analyses.

Participants who exhibited NPS in any of the five MBI domains (apathy, affective dysregulation, impulse dyscontrol, social inappropriateness, psychosis) at only one of the three visits within the first year were classified as the non-MBI NPS group, as they did not meet the MBI symptom persistence criterion. For sensitivity analyses, individuals with apathy present at only one of three visits and no persistent symptoms in other MBI domains were classified as non-MBI apathy. The no-NPS group included participants without any NPS (MBI total score = 0) at all first-year visits.

### Biomarker analysis: plasma p-tau_181_

Participants were required to have baseline plasma p-tau_181_ measurements for inclusion in this analysis. ADNI plasma p-tau_181_ concentrations (ng/L) were collected annually, with baseline blood collection occurring at or near the same scheduled study visit as the baseline NPI/NPI-Q assessment used to define initial NPS status. Samples were analyzed in a blinded manner between May and December 2019 using the Single Molecule Array (Simoa) HD-1 platform (Quanterix, Billerica, MA, USA) in the Clinical Neurochemistry Laboratory at the University of Gothenburg, Sweden, according to established protocols.^37^ The assay employs Tau12 and AT270 monoclonal antibodies to detect N-terminal to mid-domain forms of p-tau_181_. Further details on analytical procedures and assay validation are available elsewhere.^37^

### Statistical analyses

Baseline demographic characteristics comprised age, sex, and years of education. Clinical data encompassed NPI/NPI-Q and MMSE scores, and biomarker measures included apolipoprotein E4 (*APOE4*) carrier status and plasma p-tau_181_ levels. Comparisons between the MBI-apathy and non-MBI NPS group versus the no-NPS group were performed using χ^2^ tests for categorical variables and one-way ANOVAs for continuous variables.

Both cross-sectional and longitudinal associations of NPS status and plasma p-tau_181_ were examined. For the cross-sectional analysis, a linear regression model was employed with NPS status as the predictor and plasma p-tau_181_ level as the continuous outcome variable. NPS status was categorized into three groups: MBI-apathy, non-MBI NPS, and no-NPS, with the no-NPS group serving as the reference. As a sensitivity analysis, the cross-sectional model was repeated with non-MBI apathy and MBI-apathy compared against the no-NPS reference group to evaluate whether symptom persistence influenced associations with plasma p-tau_181_. The models were adjusted for age, sex, years of education, *APOE4* carrier status, and MMSE score. *APOE4* carrier status was dichotomized, with individuals carrying one or more *APOE4* alleles classified as *APOE4* carriers and those without *APOE4* alleles as non-carriers, with non-*APOE4* carriers as the reference. Male was the reference group for sex. Additional sensitivity analyses were performed to evaluate the robustness of the primary findings and are reported in the Supplemental Material.

To reduce the influence of extreme values, plasma p-tau_181_ levels were first Winsorized at the 5^th^ and 95^th^ percentiles. The Winsorized values were then log-transformed to address right-skewness before inclusion in regression analyses. The transformed distribution was then visually inspected with diagnostic plots (Residuals versus Fitted, Normal Q-Q, Scale-Location, and Residuals versus Leverage) to evaluate linearity, normality of errors, homoscedasticity, and the influence of potential outliers. The visual checks confirmed that assumptions required for regression analyses were adequately met. To facilitate interpretation, regression coefficients from the log-transformed models were exponentiated and converted to percentage differences in plasma p-tau_181_ using the formula (e^β^ − 1) × 100, where β represents the regression coefficient from the model.

Longitudinal associations between NPS status (predictor) and plasma p-tau_181_ levels (outcome variable) over two and three years were assessed using hierarchical linear mixed-effects (LME) models. Plasma p-tau_181_ levels were treated as repeated measures, whereas all other variables were assessed at baseline. To account for individual variability, participant ID was modeled with random intercepts and slopes. Fixed effects included NPS status, age, sex, years of education, APOE4 carrier status, and MMSE score. Additionally, an interaction term between NPS status and time in months was included to test whether biomarker trajectories differed across NPS groups relative to the no-NPS group.

All statistical analyses were performed using R v4.4.0. Linear models were implemented with the stats package, LME models with the lmer package, and group comparisons over time were examined using the emmeans package. To account for multiple testing and control the false discovery rate (FDR), we applied the Benjamini-Hochberg procedure to the two primary group comparisons of interest within each model (Non-MBI NPS versus no-NPS and MBI-apathy versus no-NPS). Statistical significance was defined as an adjusted *p*-value < 0.05.

## Results

### Participant demographics and characteristics

The current study included 396 participants, with 215 in the no-NPS group, 112 in the non-MBI NPS group, and 69 in the MBI-apathy group. On average, participants were 72.5 years old, 49.5% female, and 41.4% CN. In unadjusted descriptive comparisons to the no-NPS group, individuals with MBI-apathy were of similar age and education level, but were more likely to be male, *APOE4* carriers, have MCI, and higher plasma p-tau_181_ levels (Table 1).

**Table 1.** Baseline participant characteristics.

	No-NPS (n = 215)	Non-MBI NPS (n = 112)	MBI-apathy (n = 69)	*p*
Age				
Mean (SD)	72.6 (6.63)	72.4 (7.72)	72.5 (7.57)	0.982^b^
Median [Min, Max]	72.0 [56.2, 90.1]	73.0 [55.0, 88.3]	73.1 [55.0, 88.6]	
Sex				
Male	105 (48.8%)	47 (42.0%)	48 (69.6%)	**0**.**001**^a^
Female	110 (51.2%)	65 (58.0%)	21 (30.4%)	
Years of education				
Mean (SD)	16.6 (2.61)	15.9 (2.70)	16.0 (2.53)	**0**.**036**^b^
Median [Min, Max]	17.0 [12.0, 20.0]	16.0 [11.0, 20.0]	16.0 [9.00, 20.0]	
*APOE4* Carrier Status				**<0.001** ^a^
Non-*APOE4* Carrier	144 (67.0%)	68 (60.7%)	28 (40.6%)	
*APOE4* Carrier	71 (33.0%)	44 (39.3%)	41 (59.4%)	
Cognitive Status				
CN	127 (59.1%)	30 (26.8%)	7 (10.1%)	**<0**.**001**^a^
MCI	88 (40.9%)	82 (73.2%)	62 (89.9%)	
MMSE Scores				
Mean (SD)	28.8 (1.48)	28.4 (1.63)	28.0 (1.74)	**0**.**001**^b^
Median [Min, Max]	29.0 [24.0, 30.0]	29.0 [24.0, 30.0]	28.0 [24.0, 30.0]	
p-tau_181_ (ng/L)				
Mean (SD)	18.32 (32.39)	17.85 (11.36)	20.77 (11.77)	0.727^b^
Median (IQR)	13.50 (8.94, 20.57)	14.04 (10.63, 21.98)	18.17 (13.17, 24.96)	

p-tau: phosphorylated tau; APOE: Apolipoprotein E; CN: cognitively normal; MCI: mild cognitive impairment; MBI: mild behavioral impairment; MMSE: Mini-Mental State Examination; NPS: neuropsychiatric symptoms; SD: standard deviation. ^a^ χ^2^ tests, ^b^ one-way ANOVA. Boldface indicates statistically significant *p*-values (*p* < 0.05).

**Figure 2.**
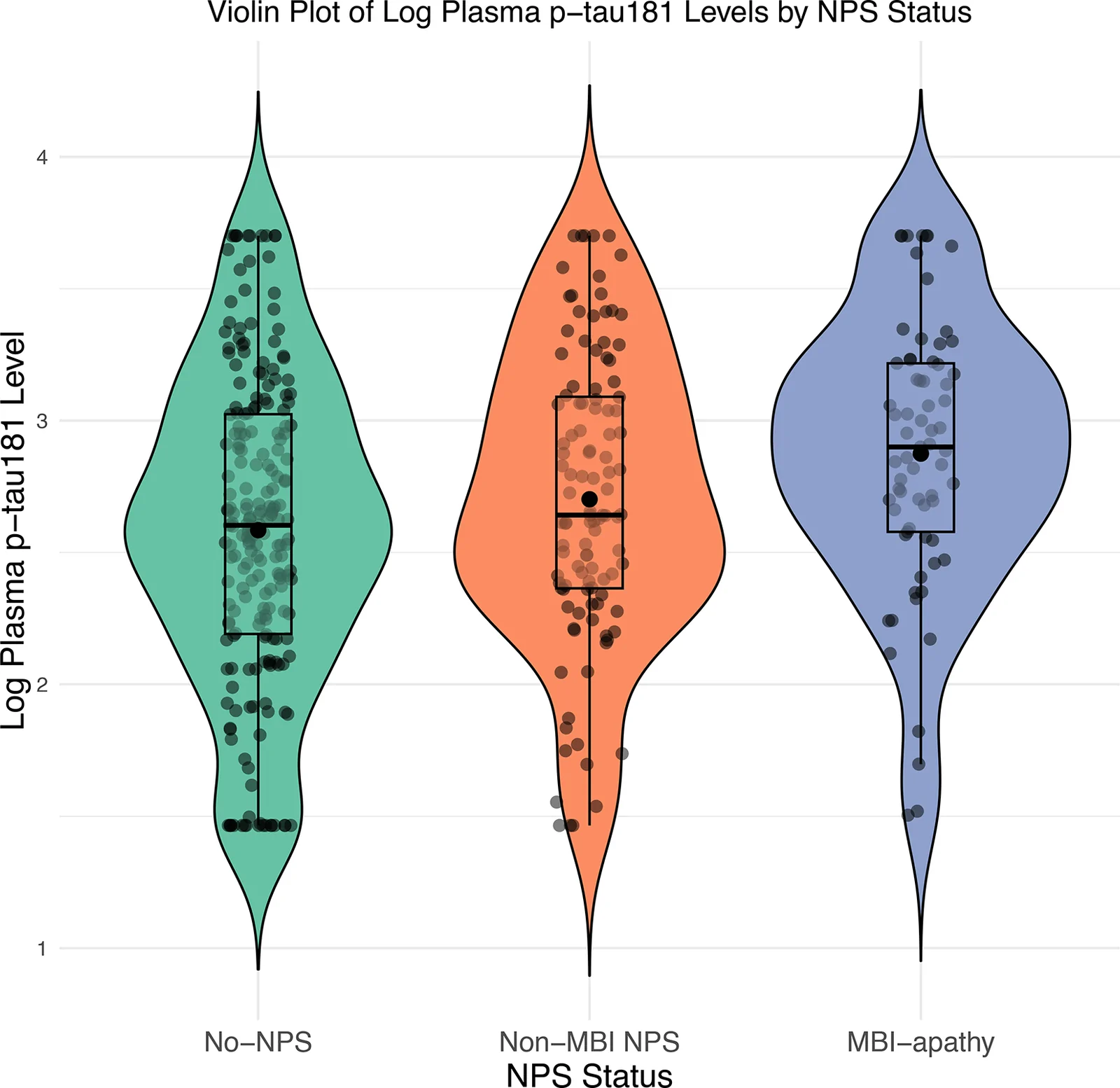
Violin plot showing the distribution of log plasma p-tau_181_ levels by NPS status, including jittered data points, boxplots for interquartile range and median, and mean values. P-tau: phosphorylated tau; APOE: apolipoprotein E; MBI: mild behavioral impairment; NPS: neuropsychiatric symptoms.

A violin plot for baseline plasma p-tau_181_ level by NPS group is presented in Figure 2.

### Cross-sectional and longitudinal associations between MBI-apathy and plasma p-Tau_181_

The cross-sectional linear regression model revealed that MBI-apathy status was associated with 24.05% higher baseline plasma p-tau_181_ levels compared to the no-NPS group (percentage difference β [95%CI], 24.05% [6.06%–45.08%]; FDR-adjusted *p* = 0.014) (Table 2). Comparatively, non-MBI NPS status was not significantly associated with plasma p-tau_181_ levels (β = 10.64% [−2.62%–25.70%]; FDR-adjusted *p* = 0.120) at baseline.

**Table 2.** Cross-sectional association between NPS group and plasma p-tau_181_ modelled using linear regression adjusted for covariates.

Predictors	%Difference in plasma p-tau_181_	95% CI	*p*	FDR-adjusted *p*
Non-MBI NPS	10.64%	−2.62%–25.70%	0.120	0.120
MBI-apathy	24.05%	6.06%–45.08%	**0**.**007**	**0**.**014**
Age	1.57%	0.76%–2.38%	**<0**.**001**	
Sex (Female)	−1.27%	−12.23%–11.06%	0.830	
Years of Education	0.50%	−1.73%–2.79%	0.663	
*APOE4* Carrier Status (*APOE4* Carrier)	23.69%	9.84%–39.28%	**<0**.**001**	
MMSE Score	−2.65%	−6.18%–1.02%	0.154	

Exponentiated regression coefficients for the NPS group variables (highlighted in grey) represent the estimated percent difference in p-tau_181_ compared to the no-NPS group at baseline. This model is adjusted for age, sex, years of education, *APOE4* carrier status, and MMSE score. *APOE*: Apolipoprotein E; CI: confidence interval; FDR: false discovery rate; MBI: mild behavioral impairment; MMSE: Mini-Mental State Examination; NPS: neuropsychiatric symptoms. Boldface indicates statistically significant *p*-values (*p* < 0.05).

In the sensitivity analysis, non-MBI apathy was not associated with plasma p-tau_181_, whereas MBI-apathy remained significantly associated with higher plasma p-tau_181_ levels (Supplemental Table 1). Additional sensitivity analyses are reported in Supplemental Tables 2–6.

**Figure 3.**
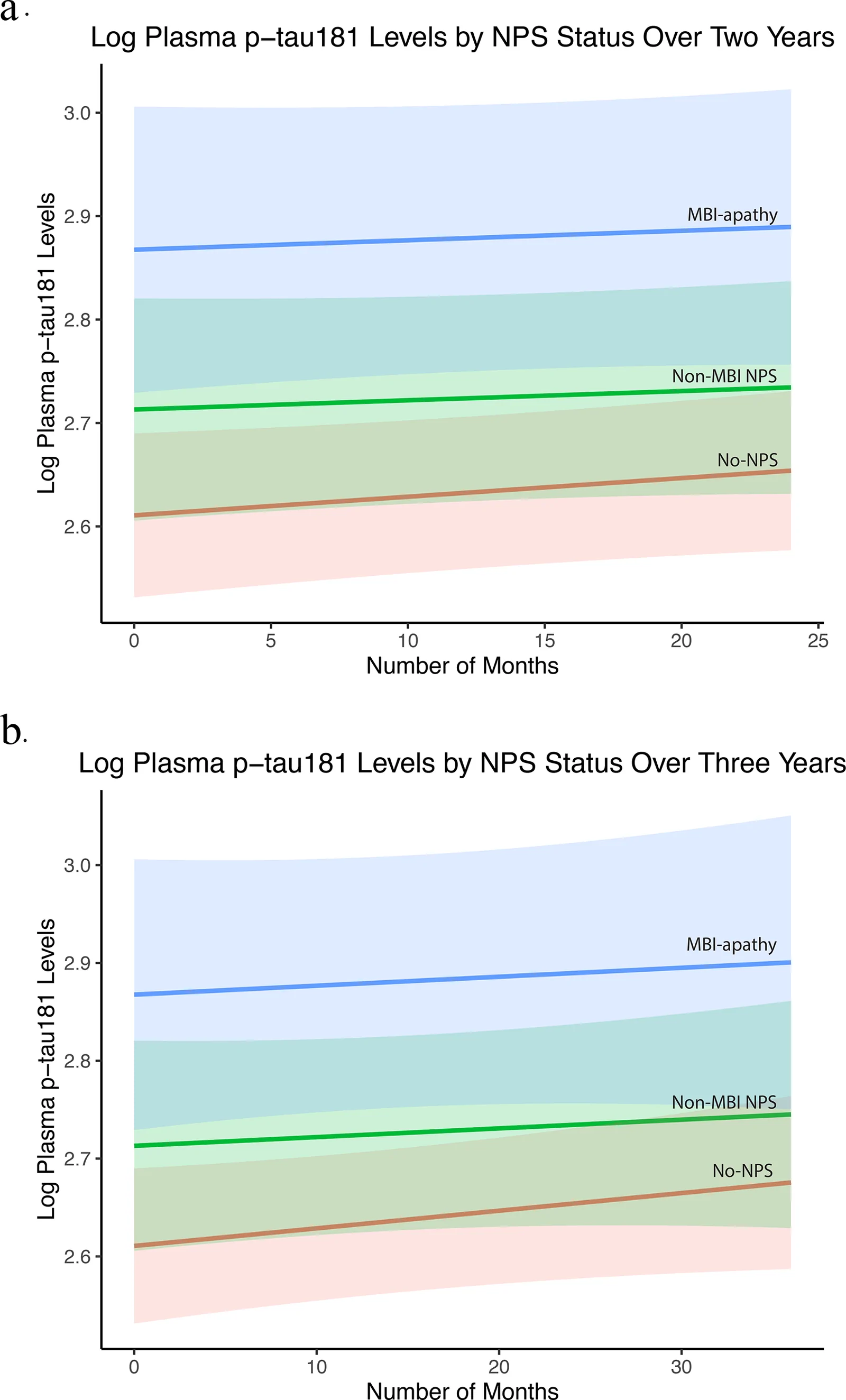
Longitudinal trajectories of plasma p-tau_181_ over time stratified by NPS status. Panels display estimated marginal mean trajectories with 95% confidence intervals for a. p-tau_181_ over two years and b. plasma p-tau_181_ over three years, stratified by NPS status: MBI-apathy, non-MBI NPS, and no-NPS (reference group). p-tau: phosphorylated tau; MBI: mild behavioral impairment; NPS: neuropsychiatric symptoms.

Longitudinal biomarker data for plasma p-tau_181_ were available for 328/396 participants at the two-year mark (Table 3 and Figure 3).

**Table 3.** Longitudinal association between NPS group and plasma p-tau_181_ over two years modelled using linear mixed effect models adjusted for covariates.

Predictors	%Difference in plasma p-tau_181_	95% CI	*p*	FDR-adjusted *p*
Non-MBI NPS	11.18%	−2.84%–27.23%	0.126	0.126
MBI-apathy	26.46%	7.24%–49.12%	**0**.**006**	**0**.**012**
Time (Months)	0.15%	−0.14%–0.43%	0.318	
Age	1.96%	1.17%–2.75%	**<0**.**001**	
Sex (Female)	−5.77%	−15.92%–5.60%	0.310	
Years of Education	0.12%	−2.04%–2.33%	0.914	
*APOE4* Carrier Status (*APOE4* Carrier)	22.23%	8.93%–37.15%	**0**.**001**	
MMSE Score	−2.53%	−5.96%–1.03%	0.165	
NPS status (Non-MBI NPS) × Time	−0.14%	−0.63%–0.35%	0.580	
NPS status (MBI-apathy) × Time	0.22%	−0.38%–0.82%	0.479	

Exponentiated regression for the NPS group variables (highlighted in grey) represent the estimated percent difference in p-tau_181_ compared to the no-NPS group over two years. Tests of interaction between NPS group and time were non-significant. This model is adjusted for age, sex, years of education, *APOE4* carrier status, MMSE score, and an interaction term between NPS status and number of months. *APOE*: Apolipoprotein E; CI: confidence interval; FDR: false discovery rate; MBI: mild behavioral impairment; MMSE: Mini-Mental State Examination; NPS: neuropsychiatric symptoms. Boldface indicates statistically significant *p*-values (*p* < 0.05).

Participants missing plasma p-tau_181_ data at two years were older (74.63 [6.77] versus 72.05 [7.10] years, *p* = 0.005) and had lower MMSE scores (28.03 [1.88] versus 28.62 [1.51], *p* = 0.017) compared to those with data available at that timepoint. Sex distribution (Female: 52.94% versus 48.78%, *p* = 0.623), years of education (15.79 ± 2.86 versus 16.44 ± 2.58, *p* = 0.087), and *APOE4* carriership (48.53% versus 37.50%, *p* = 0.119) did not significantly differ between groups. On average, plasma p-tau_181_ levels did not significantly change over two years across the full sample (percentage change β [95%CI], 0.15% [−0.14%–0.43%]; *p* = 0.318). However, two-year hierarchal LME analyses revealed significant between-group differences. Compared to the no-NPS group, MBI-apathy status was associated with significantly higher p-tau_181_ levels over two years (26.46% [7.24%–49.12%]; FDR-adjusted *p* = 0.012), while non-MBI NPS status was not (11.18% [−2.84%–27.23]; FDR-adjusted *p* = 0.126). Tests of interactions between NPS group and time were not statistically significant (*p* > 0.05), suggesting that, within this two-year period, there were no detectable differences in the slopes of change in p-tau_181_ between the groups.

Longitudinal biomarker data for plasma p-tau_181_ were available for 186/396 participants at the three-year mark (Table 4 and Figure 3).

**Table 4.** Longitudinal association between NPS group and plasma p-tau_181_ over three years modelled using linear mixed effect models adjusted for covariates.

Predictors	%Difference in plasma p-tau_181_	95% CI	*p*	FDR-adjusted *p*
Non-MBI NPS	10.78%	−2.81%–26.27%	0.128	0.128
MBI-apathy	29.28%	10.17%–51.72%	**0**.**002**	**0**.**004**
Time (Months)	0.18%	−0.04%–0.40%	0.108	
Age	1.94%	1.17%–2.71%	**<0**.**001**	
Sex (Female)	−4.61%	−14.66%–6.64%	0.411	
Years of Education	0.05%	−2.06%–2.21%	0.961	
*APOE4* Carrier Status (*APOE4* Carrier)	22.58%	9.52%–37.19%	**<0**.**001**	
MMSE Score	−2.69%	−6.04%–0.78%	0.130	
NPS status (Non-MBI NPS) × Time	−0.09%	−0.45%–0.26%	0.617	
NPS status (MBI-apathy) × Time	−0.09%	−0.51%–0.33%	0.680	

Exponentiated regression for the NPS group variables (highlighted in grey) represent the estimated percent difference in p-tau_181_ compared to the no-NPS group over three years. Tests of interaction between NPS group and time were non-significant. This model is adjusted for age, sex, years of education, *APOE4* carrier status, MMSE score, and an interaction term between NPS status and number of months. *APOE*: Apolipoprotein E; CI: confidence interval; FDR: false discovery rate; MBI: mild behavioral impairment; MMSE: Mini-Mental State Examination; NPS: neuropsychiatric symptoms. Boldface indicates statistically significant *p*-values (*p* < 0.05).

Participants missing plasma p-tau_181_ data at three years were older (73.60 [6.70] versus 71.25 [7.36] years, *p* = 0.001) and had higher MMSE scores (28.84 [1.45] versus 28.15 [1.67], *p* < 0.001) compared to those with data available at that timepoint. Sex distribution (Female: 46.67% versus 52.69%, *p* = 0.273), years of education (16.32 [2.64] versus 16.34 [2.64], *p* = 0.925), and *APOE4* carriership (35.71% versus 43.55%, *p* = 0.136) did not significantly differ between groups. On average, plasma p-tau_181_ did not significantly change over three years across the full sample (0.18% [−0.04%–0.40%]; *p* = 0.108). However, three-year hierarchal LME analyses revealed that compared to the No-NPS group, MBI-apathy was associated with higher p-tau_181_ over three years (29.28% [10.17%–51.72%]; FDR-adjusted *p* = 0.004), while non-MBI NPS was not (10.78% [−2.81%–26.27]; FDR-adjusted *p* = 0.128). Tests of interactions between NPS group and time were not statistically significant (*p* > 0.05), suggesting that, within the limits of our study, there are no detectable differences in the slopes of changes in p-tau_181_ between the groups.

## Discussion

In this study of 396 dementia-free older adults, we examined associations between MBI-apathy and blood plasma p-tau_181_ levels. Associations were assessed cross-sectionally and longitudinally over two and three years, comparing individuals with MBI-apathy and non-MBI NPS to those without NPS. Apathy symptoms meeting MBI criteria were consistently associated with higher plasma p-tau_181_ levels at baseline and follow-up, whereas non-MBI NPS showed no significant association.

Prior work from our group showed that, in CN and MCI individuals, MBI-apathy was associated with greater CSF tau pathology.^27^ In ADNI, individuals with MBI-apathy exhibited significantly higher CSF p-tau_181_ and CSF p-tau_181_/Aβ_42_ at baseline and over two years, while non-MBI NPS did not significantly differ from no-NPS. A four-year analysis in ADNI and MEMENTO cohorts supports this pattern at the global MBI level, although apathy was not examined specifically.^38^ Across four years, MBI was associated with higher CSF p-tau_181_ and CSF p-tau_181_/Aβ_42_, while non-MBI NPS showed no significant association. More recently, MBI has also been significantly associated with CSF Aβ_42_ and p-tau_181_ positivity, as well as with the NIA-AA defined AD continuum biomarker profile, whereas non-MBI NPS showed no associations.^39^ Concordant results were obtained with plasma markers for p-tau_181_ and p-tau_217_.^40,41^ Global MBI was associated with significantly higher plasma p-tau_181_ at baseline and over four years, while non-MBI NPS again showed no difference.^40^ Global MBI was also significantly associated with higher plasma p-tau_217_ at baseline compared to those without MBI.^41^ Similarly, higher MBI Checklist (MBI-C) scores have been associated with both elevated CSF p-tau_181_ and higher tau-PET signal in the entorhinal cortex and hippocampus among CN Aβ-positive individuals.^42^ Extending these findings, MBI status has been associated with greater tau tracer uptake in early AD cortical regions among Aβ-positive but not Aβ-negative individuals, reinforcing its role as a behavioral correlate of AD-related tau pathology.^43^ These biomarker results align with autopsy studies showing that MBI in the five years before death is significantly associated with pathologically confirmed AD, but not with non-AD neuropathologies, underscoring its specificity as a prodromal feature of AD.^44,45^

Together, these findings show that applying the MBI framework to all later-life NPS helps identify the NPS subgroup most likely to have tau-related neurodegeneration. In the present study, individuals with MBI-apathy had the highest levels of plasma p-tau_181_, supporting the potential utility of this behavioral phenotype for identifying preclinical and prodromal stages of the AD continuum. A key MBI feature is symptom persistence. Across all cohorts, elevated tau was observed only when symptoms had later-life onset and persisted for at least six months. In contrast, impersistent NPS, such as those captured in the non-MBI NPS group, can reflect situational, psychological, or comorbid medical factors unrelated to neurodegeneration.^18^ Pooling impersistent with persistent symptoms weakens observed biomarker associations, while excluding episodic cases sharpens the signal. In practice, applying MBI criteria does more than refine a case definition, it isolates a behavioral phenotype that more reliably indexes early AD pathology. Therefore, future biomarker studies and clinical trials should distinguish MBI from non-MBI NPS, as these groups differ in likelihood of underlying AD pathology. While most biomarker studies have examined MBI globally, the present findings highlight the importance of considering domain-specific behavioral phenotypes. In particular, the association between MBI-apathy and plasma p-tau_181_ supports prior evidence suggesting that apathy may be especially closely linked to AD-related pathology. Building on our previous CSF findings in the same cohort,^27^ the current results extend this domain-specific relationship to plasma tau biomarkers, revealing that persistent apathy may represent a behavioral signal of underlying AD pathology detectable using scalable blood-based measures.

Plasma p-tau_181_ levels remained higher in the MBI-apathy group over time; however, our longitudinal LMEs did not identify significant interaction effects between NPS group and time. As shown in Figure 3, the MBI-apathy group had roughly parallel trajectories to the other groups. The non-significant interaction suggests that, within the study timeframe, group trajectories in p-tau_181_ remained relatively stable and did not diverge significantly. The observed stability is consistent with AD biomarker staging models, which suggest that plasma p-tau_181_ rises after detectable amyloid accumulation and begins to plateau during late-MCI to early-dementia.^1,5,11,37^ In contrast, longitudinal data from sporadic and autosomal dominant cohorts show that p-tau_217_ increases earlier in the AD cascade.^1,4,5,11,46^ As p-tau_181_ increases later in the disease course, it may better reflect downstream progression rather than initial onset. The absence of a significant interaction however, does not necessarily confirm identical slopes across groups. Absence of slope differences may indicate a biological inflection point occurring before our baseline. This inflection point coincides with global MBI, the construct for which was developed explicitly for early detection; MBI can initially manifest as the emergence and persistence of an undifferentiated constellation of symptoms prior to an identifiable MBI domain.^27,40,47^ Group-level differences in p-tau_181_ trajectories may therefore have already stabilized by enrolment, limiting our ability to detect divergence over the observed two- to three-year period. Short follow-up duration, modest assay sensitivity for detecting subtle change in plasma p-tau_181_, inter-individual variability, and attrition-related reductions in sample size could have further contributed to the absence of slope differences. Nonetheless, the consistently elevated p-tau_181_ levels observed in the MBI-apathy group, independent of trajectory acceleration, underscores its potential utility as a behavioral marker of early tau pathology. Within this context, emerging neuropathological evidence suggests that early tau changes may precede detectable amyloid accumulation, particularly in brainstem nuclei, such as the locus coeruleus, highlighting the need for future studies integrating emerging biomarkers of early tau species to further refine current models of AD pathophysiology.^48–50^

The elevated risk profile of the MBI-apathy group is further underscored by its demographic characteristics. In our sample, those with MBI-apathy were more likely to carry the *APOE4* allele, have MCI, and be male, features that, while adjusted for in all models, highlight clinical differences that may contribute to the early identification of at-risk individuals. These findings align with prior work from our group. For example, in a National Alzheimer's Coordinating Center analysis, MBI-apathy cases were more often male (65.0%) and had higher MCI prevalence (58.5%) than those without apathy (39.7% male, 20.0% with MCI) and no NPS (37.4% male, 13.4% with MCI).^24^ Similarly, in our ADNI CSF biomarker study, more *APOE4* carriers (59.6%) and males (67.3%) were observed among those with MBI-apathy compared to those with no NPS (48.8% male, 33.0% *APOE4* carriers) and non-MBI NPS (45.7% male, 31.9% *APOE4* carriers).^27^ Although male sex is not a risk factor for AD, and women have a higher lifetime risk,^51^ several studies suggest that apathy may be more common or severe in males.^19,52,53^ A meta-analysis showed that across AD clinical stages, apathy severity was higher in men, in contrast to depression or anxiety, which were more prevalent in women.^52^ Sex differences may reflect differential involvement of fronto-striatal circuits or dopaminergic tone, both of which are implicated in apathy.^54^ Differences in symptom reporting or recognition may also contribute, as men may be less likely to report affective complaints, instead presenting with motivational withdrawal. In addition, study partner characteristics may shape symptom detection and endorsement. Female study partners are more likely than male study partners to report NPS, which may influence observed sex differences.^55^ Taken together, these demographic patterns suggest that MBI-apathy clusters with other established indicators of elevated AD risk. Understanding the intersection of behavioral, genetic, and cognitive features could improve our ability to stratify risk and tailor early intervention strategies.

### Limitations

This study has a few relevant limitations that should be mentioned. First, the ADNI dataset is derived from a highly curated research cohort that was designed to be enriched for AD pathology. As such, participants are selected based on strict criteria, including minimal vascular burden and elevated risk of AD, making the cohort less representative of the general aging population. Additionally, the sample has limited ethnocultural and sociodemographic diversity, with participants predominantly identifying as White and very small cell counts for other ethnicity categories, further restricting generalizability and precluding meaningful subgroup analyses by ethnicity.

The present study was designed as a hypothesis-driven analysis focused specifically on MBI-apathy, based on prior findings showing an association with CSF p-tau_181_.^27^ However, the other individual MBI domains were not examined independently in relation to plasma p-tau_181_. While this study supports an association between MBI-apathy and plasma p-tau_181_, it does not imply that apathy is the only MBI domain associated with this biomarker. Examining all individual MBI domains would require adequately powered analyses with appropriate consideration of multiple comparisons. Future studies are needed to determine associations between persistent behavioral changes and plasma tau biomarkers across all MBI domains.

The MBI-apathy group is relatively small. While this was anticipated given the specificity of the MBI criteria, it limits our ability to examine more nuanced relationships. For instance, we were unable to assess interactions between NPS status and clinical cognitive diagnosis (i.e., CN versus MCI), as the number of CN individuals with MBI-apathy was too small for meaningful stratification (n = 5 at two years; n = 1 at three years). In addition, in a sensitivity analysis replacing MMSE with CDR Sum of Boxes (CDR-SB), the association between MBI-apathy and plasma p-tau_181_ was attenuated and no longer statistically significant (Supplemental Table 2). Since CDR-SB captures cognitive and functional impairment, this finding suggests that the observed association may be partly influenced by clinical disease severity and highlights the need for replication in larger samples that can more fully account for cognitive and functional status. The limited sample also precluded analysis of plasma biomarkers beyond p-tau_181_, including p-tau_217_, despite growing evidence supporting p-tau_217_ as a more sensitive and diagnostically specific marker of AD pathology, including improved differentiation of AD from other neurodegenerative conditions.^3,11–17^ Moreover, while plasma p-tau_181_ is an AD-relevant biomarker, elevated levels are not sufficient in isolation to establish AD-specific pathology or distinguish AD from other neurodegenerative conditions, including frontotemporal dementia (FTD). MBI is intended to identify increased dementia risk rather than to establish a specific underlying neurodegenerative etiology. This consideration is especially relevant when examining apathy, which can occur across neurodegenerative disorders, including behavioral variant FTD.^30^ Accordingly, the observed association between MBI-apathy and plasma p-tau_181_ should be interpreted as suggestive of alignment with AD-related tau pathology in this sample rather than as evidence that MBI-apathy specifically identifies AD. Indeed, our research group has recently found an association between MBI and plasma p-tau_217_,^41^ highlighting the need for future studies incorporating more informative AD biomarkers, including plasma p-tau_217_ and confirmatory amyloid or tau biomarkers, alongside biomarkers of other dementia etiologies, to better characterize the underlying neurodegenerative pathology in individuals with MBI-apathy.

In addition, apathy was operationalized using the NPI and NPI-Q, instruments originally developed for dementia populations. These tools may have limited sensitivity for detecting subtle or early motivational changes in non-dementia individuals, particularly when using the brief NPI-Q, potentially underestimating apathy severity or prevalence in preclinical stages. Future studies using the MBI-C might be informative, as the assessment explicitly identifies apathy and the three apathy domains of interest, initiative, and emotional reactivity, and may be better suited for examining variability in behavioral trajectories and their relationship to underlying AD pathology.^56–58^

Additionally, plasma p-tau_181_ was analyzed as a continuous outcome in this study, allowing detection of subtle group-level differences but not whether individuals with MBI-apathy were more likely to exceed clinical or pathological cutoffs. Future research incorporating binary classifications of biomarker positivity (e.g., normal versus abnormal plasma p-tau_181_) and comparison with non-MBI apathy could further clarify the prognostic relevance of persistent, later-life emergent apathy for risk stratification and early detection. Finally, our longitudinal analyses relied on LME models, which assume linear trajectories. Although diagnostic checks supported model adequacy, it is possible that non-linear changes in plasma p-tau_181_ were not fully captured. Future studies with larger samples and extended follow-up could examine flexible modeling approaches to better account for potential non-linearity.

### Conclusions

MBI-apathy represents a clinically meaningful behavioral syndrome that aligns with biological and cognitive markers of AD risk. In this study, emergent and persistent apathy symptoms, defined using the MBI framework, were associated with elevated plasma p-tau_181_, reinforcing the link between later-life behavioral change and early tau pathology. These findings build on a growing body of research supporting the utility of MBI for sample enrichment and risk stratification in preclinical and prodromal AD.

As blood-based biomarkers gain traction in both research and clinical settings, incorporating scalable behavioral indicators, such as MBI-apathy, offers a complementary and pragmatic approach to early detection. Relevance is heightened in contexts where biomarker access remains limited or cost-prohibitive. Screening for emergent and persistent apathy symptoms may help identify individuals who would benefit from closer monitoring, timely counselling, or preventative intervention. Future research should continue to explore how integrating behavioral and biological data can improve prognostic accuracy and inform targeted, stage-specific care strategies in AD.

## Supplemental Material

sj-docx-1-alz-10.1177_13872877261470257 - Supplemental material for Mild behavioral impairment-apathy and Alzheimer's disease plasma phosphorylated tau biomarker levels Supplemental material, sj-docx-1-alz-10.1177_13872877261470257 for Mild behavioral impairment-apathy and Alzheimer's disease plasma phosphorylated tau biomarker levels by Daniella Vellone, Rebeca Leon, Zahra Goodarzi, Nils D. Forkert, Eric E. Smith and Zahinoor Ismail in Journal of Alzheimer's Disease
