# Supplementary material for: Mild behavioral impairment-apathy and Alzheimer's disease plasma phosphorylated tau biomarker levels
Source: J Alzheimers Dis. 2026 Jul 29;113(2):614–27. doi: 10.1177/13872877261470257 (PMC13554361; doi:10.1177/13872877261470257)
Supplement: Supplementary file 1 [file sj-docx-1-alz-10.1177_13872877261470257.docx]

**Supplemental Material**

**Mild behavioral impairment-apathy and Alzheimer’s disease plasma phosphorylated tau biomarker levels**

**Supplemental Table 1.** Cross-sectional association between NPS group (including Non-MBI apathy) and plasma p-tau_181_ modelled using linear regression adjusted for covariates.

| **Predictors** | **%Difference in Plasma p-tau181** | **95% CI** | ***p*** |
| --- | --- | --- | --- |
| Non-MBI apathy | -9.96% | -32.69% – 20.44% | 0.478 |
| MBI-apathy | 21.84% | 4.21% – 42.45% | **0.013** |
| Age | 0.86% | -0.07% – 1.80% | 0.070 |
| Sex (Female) | -2.44% | -14.66% – 11.52% | 0.716 |
| Years of Education | 0.70% | -1.90% – 3.36% | 0.600 |
| *APOE4* Carrier Status (*APOE4* Carrier) | 19.39% | 4.11% – 36.90% | **0.011** |
| MMSE Score | - 4.95% | -8.91% – 0.82% | **0.019** |

Exponentiated regression coefficients for the NPS group variables (highlighted in grey) represent the estimated percent difference in p-tau_181_ compared to the no-NPS group at baseline. This model is adjusted for age, sex, years of education, *APOE4* carrier status, and MMSE score. *APOE*: Apolipoprotein E; CI: confidence interval; MBI: mild behavioral impairment; MMSE: Mini-Mental State Examination; NPS: neuropsychiatric symptoms.

**Supplemental Table 2.** Cross-sectional association between NPS group and plasma p-tau_181_ in a model adjusting for CDR-SB in place of the MMSE.

| **Predictors** | **%Difference in Plasma p-tau_181_** | **95% CI** | ***p*** |
| --- | --- | --- | --- |
| Non-MBI NPS | 8.87% | -4.44% – 24.04% | 0.201 |
| MBI-apathy | 15.65% | -4.15% – 39.54% | 0.129 |
| Age | 1.69% | 0.89% – 2.49% | **<0.001** |
| Sex (Female) | -1.96% | -12.84% – 10.26% | 0.740 |
| Years of Education | 0.32% | -1.88% – 2.56% | 0.778 |
| *APOE4* Carrier Status (*APOE4* Carrier) | 24.14% | 10.39% – 39.60% | **<0.001** |
| CDR-SB Score | 5.88% | -1.50% – 13.81% | 0.121 |

Exponentiated regression coefficients for the NPS group variables (highlighted in grey) represent the estimated percentage difference in plasma p-tau_181_ compared to the no-NPS group at baseline. This sensitivity analysis replaced the MMSE with CDR-SB to account for cognitive and functional disease severity. The model was adjusted for age, sex, years of education, *APOE4* carrier status, and CDR-SB score. *APOE*: Apolipoprotein E; CDR-SB: Clinical Dementia Rating Sum of Boxes; CI: confidence interval; MBI: mild behavioral impairment; MMSE: Mini-Mental State Examination; NPS: neuropsychiatric symptoms.

**Supplemental Table 3.** Cross-sectional association between NPS group and plasma p-tau_181_ in a model adjusting for RAVLT percentage forgetting score in place of MMSE.

| **Predictors** | **%Difference in Plasma p-tau181** | **95% CI** | ***p*** |
| --- | --- | --- | --- |
| Non-MBI NPS | 10.14% | -3.09% – 25.19% | 0.139 |
| MBI-apathy | 22.99% | 5.01% – 44.04% | **0.010** |
| Age | 1.58% | 0.78% – 2.39% | **<0.001** |
| Sex (Female) | -0.10% | -11.33% – 12.54% | 0.986 |
| Years of Education | 0.39% | -1.82% – 2.65% | 0.730 |
| *APOE4* Carrier Status (*APOE4* Carrier) | 23.85% | 10.07% – 39.36% | **<0.001** |
| RAVLT Percentage Forgetting Score | 0.14% | -0.04% – 0.32% | 0.125 |

Exponentiated regression coefficients for the NPS group variables (highlighted in grey) represent the estimated percentage difference in plasma p-tau_181_ compared to the no-NPS group at baseline. This sensitivity analysis replaced the MMSE with the RAVLT percentage forgetting score as a measure of episodic memory performance. The model was adjusted for age, sex, years of education, *APOE4* carrier status, and RAVLT percentage forgetting score. *APOE*: Apolipoprotein E; CI: confidence interval; MBI: mild behavioral impairment; MMSE: Mini-Mental State Examination; NPS: neuropsychiatric symptoms; RAVLT: Rey Auditory Verbal Learning Test.

**Supplemental Table 4.** Cross-sectional association between NPS group and plasma p-tau_181_ using upper-tail-only Winsorization.

| **Predictors** | **%Difference in Plasma p-tau_181_** | **95% CI** | ***p*** |
| --- | --- | --- | --- |
| Non-MBI NPS | 13.64% | -1.44% – 31.02% | 0.078 |
| MBI-apathy | 28.24% | 7.68% – 52.73% | **0.005** |
| Age | 1.66% | 0.75% – 2.57% | **<0.001** |
| Sex (Female) | -0.31% | -12.57% – 13.67% | 0.962 |
| Years of Education | 0.72% | -1.78% – 3.27% | 0.577 |
| *APOE4* Carrier Status (*APOE4* Carrier) | 26.54% | 10.84% – 44.46% | **<0.001** |
| MMSE Score | -3.30% | -7.20% – 0.77% | 0.111 |

Exponentiated regression coefficients for the NPS group variables (highlighted in grey) represent the estimated percentage difference in plasma p-tau_181_ compared to the no-NPS group at baseline. In this sensitivity analysis, plasma p-tau_181_ values were Winsorized at the 95^th^ percentile only and subsequently log-transformed before regression modelling. The model was adjusted for age, sex, years of education, *APOE4* carrier status, and MMSE score. *APOE*: Apolipoprotein E; CI: confidence interval; MBI: mild behavioral impairment; MMSE: Mini-Mental State Examination; NPS: neuropsychiatric symptoms.

**Supplemental Table 5.** Cross-sectional association between NPS group and plasma p-tau_181_ after excluding participants who converted from MCI to dementia during the first year

| **Predictors** | **%Difference in Plasma p-tau181** | **95% CI** | ***p*** |
| --- | --- | --- | --- |
| Non-MBI NPS | 12.52% | -2.83% – 30.31% | 0.115 |
| MBI-apathy | 24.78% | 2.81% – 51.44% | **0.025** |
| Age | 1.69% | 0.74% – 2.64% | **<0.001** |
| Sex (Female) | 1.13% | -11.81% – 15.97% | 0.872 |
| Years of Education | 0.81% | -1.82% – 3.51% | 0.550 |
| *APOE4* Carrier Status (*APOE4* Carrier) | 23.87% | 7.83% – 42.28% | **0.003** |
| MMSE Score | -2.51% | -6.68% – 1.85% | 0.254 |

Exponentiated regression coefficients for the NPS group variables (highlighted in grey) represent the estimated percentage difference in plasma p-tau_181_ compared with the no-NPS group at baseline. This sensitivity analysis excluded participants who converted from MCI to dementia during the first-year period. The model was adjusted for age, sex, years of education, *APOE4* carrier status, and MMSE score. *APOE*: Apolipoprotein E; CI: confidence interval; MBI: mild behavioral impairment; MCI: mild cognitive impairment; MMSE: Mini-Mental State Examination; NPS: neuropsychiatric symptoms.

**Supplemental Table 6.** Cross-sectional association between NPS group and plasma p-tau_181_ restricted to participants with MCI.

| **Predictors** | **%Difference in Plasma p-tau181** | **95% CI** | ***p*** |
| --- | --- | --- | --- |
| Non-MBI NPS | 8.70% | -8.02% – 28.46% | 0.326 |
| MBI-apathy | 26.03% | 4.70% – 51.71% | **0.015** |
| Age | 1.63% | 0.66% – 2.61% | **0.001** |
| Sex (Female) | -5.66% | -19.30% – 10.28% | 0.463 |
| Years of Education | -0.76% | -3.56% – 2.13% | 0.603 |
| *APOE4* Carrier Status (*APOE4* Carrier) | 25.82% | 8.63% – 45.72% | **0.002** |

Exponentiated regression coefficients for the NPS group variables (highlighted in grey) represent the estimated percentage difference in plasma p-tau_181_ compared to the no-NPS group at baseline among participants with MCI. The model was adjusted for age, sex, years of education, and *APOE4* carrier status. *APOE*: Apolipoprotein E; CI: confidence interval; MBI: mild behavioral impairment; MCI: mild cognitive impairment; NPS: neuropsychiatric symptoms.
